# An Extremely Rare Case of a Primary Pancreatic Yolk Sac Tumor

**DOI:** 10.7759/cureus.26007

**Published:** 2022-06-16

**Authors:** Ilias Galanis, Georgios Floros, Magdalini Simou, Georgios Kyriakopoulos, Georgios Stylianidis

**Affiliations:** 1 2nd Department of Surgery, Evaggelismos General Hospital, Athens, GRC; 2 Department of Pathology, Evaggelismos General Hospital, Athens, GRC

**Keywords:** pancreatic tumor, extragonadal germ cell tumor, pancreas, germ cell tumor, yolk sac tumor

## Abstract

Yolk sac tumor (YST) is a rare malignant type of germ cell tumor (GCT). Extragonadal yolk sac tumor is a very rare entity. We report the case of a 33-year-old male with a pancreatic mass, which proved to be a primary yolk sac tumor, arising in the pancreas.

## Introduction

Yolk sac tumors (YSTs) are rare and highly malignant germ cell tumors (GCTs) [1]. GCTs consist of a wide variety of tumors with teratomas being the most common and YSTs being the most common malignant type of GCTs [2]. YSTs are mostly found in infants and young adolescents, with a median age of 19 years. Primary YSTs occur in the testis or ovary, and only 3-5% may arise in a variety of midline extragonadal sites, such as, in decreasing order of frequency, the mediastinum, retroperitoneum, sacrococcygeal region and pineal gland [3]. The explanation of this misplacement remains controversial. There are two main theories trying to explain the existence of primary extragonadal GCTs, comprised either of the abnormal differentiation of somatic cells or the misplacement of germ cells during embryogenesis [4,5]. To our knowledge, primary YSTs of the pancreas, especially in a 33-year-old patient, are extremely rare with very few cases reported in the literature.

## Case presentation

**Figure 1:**
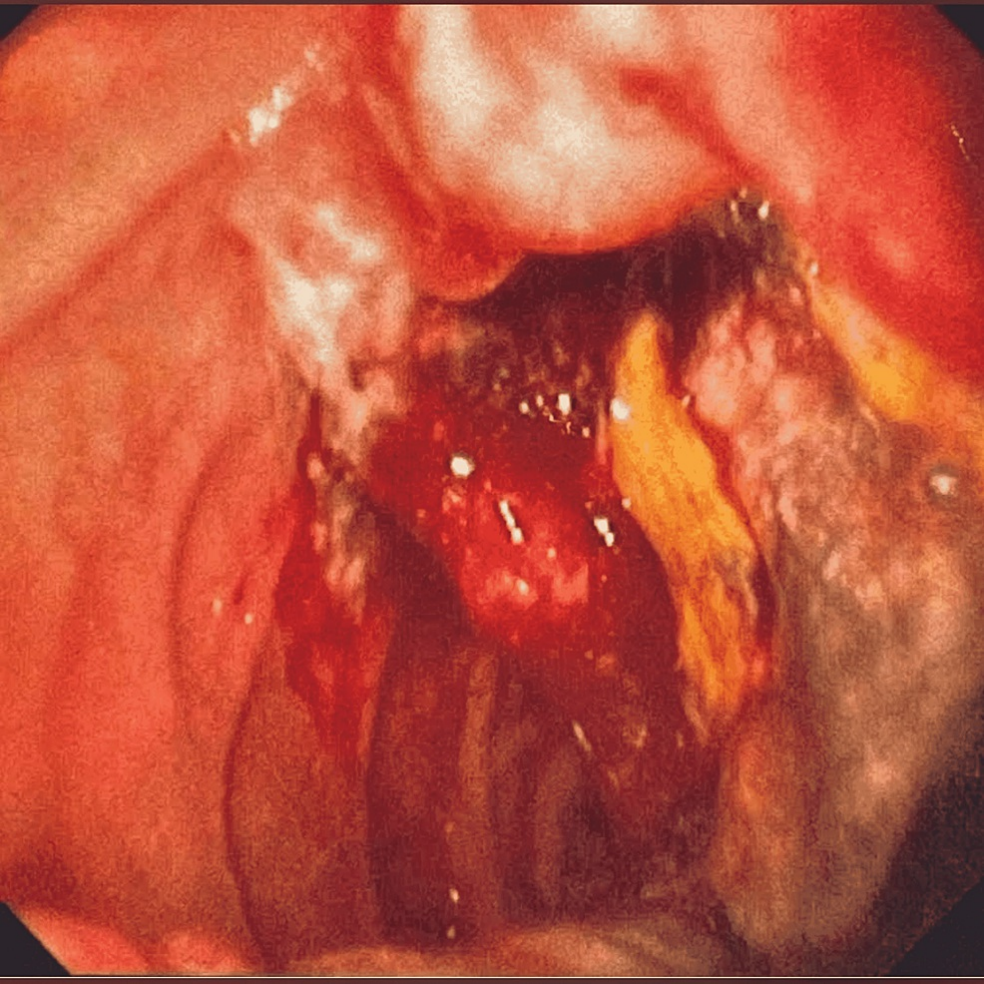
Ulcerated lesion of the second part of the duodenum.

**Figure 2:**
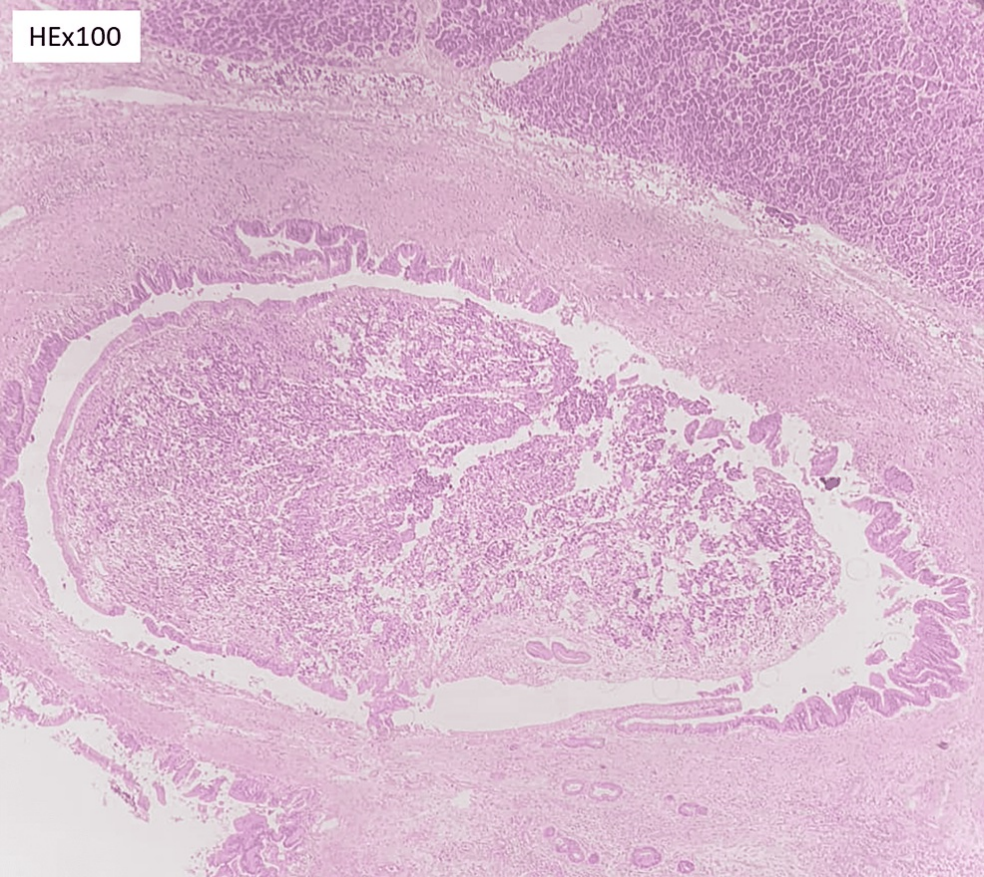
Intraluminal growth of neoplastic tumor in the main pancreatic duct

**Figure 3:**
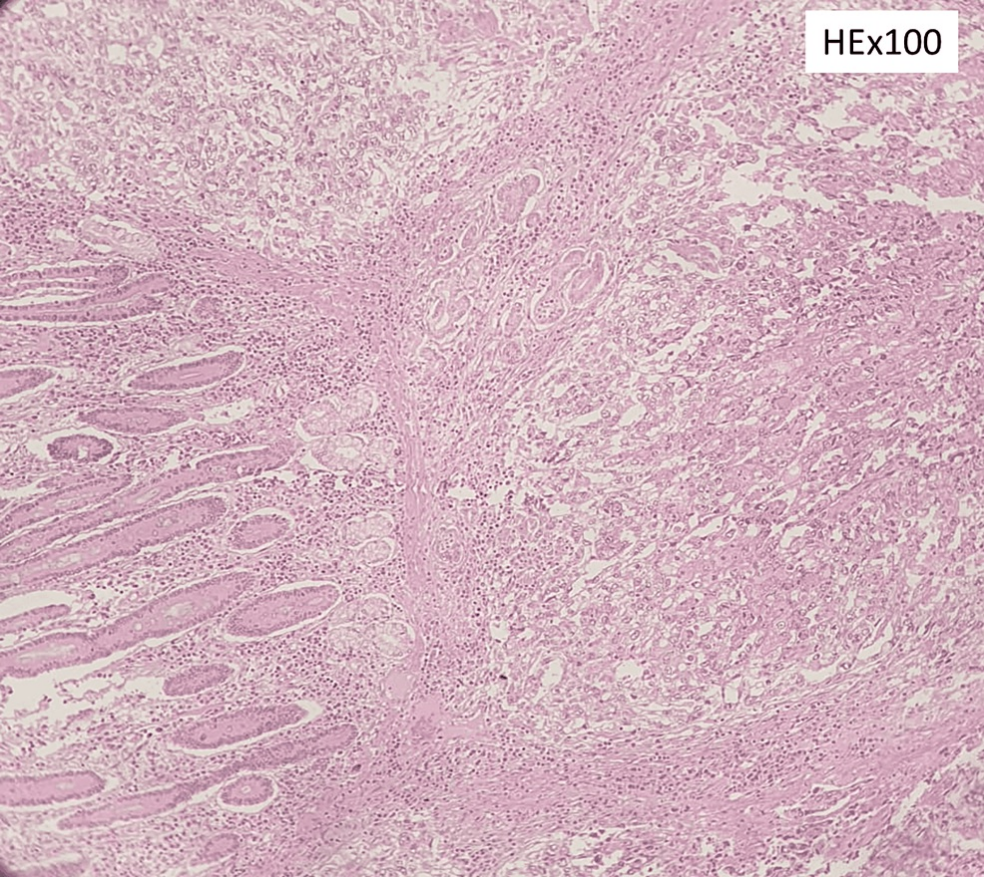
Invasive growth pattern and extension of neoplastic tumor in the duodenal mucosa

**Figure 4:**
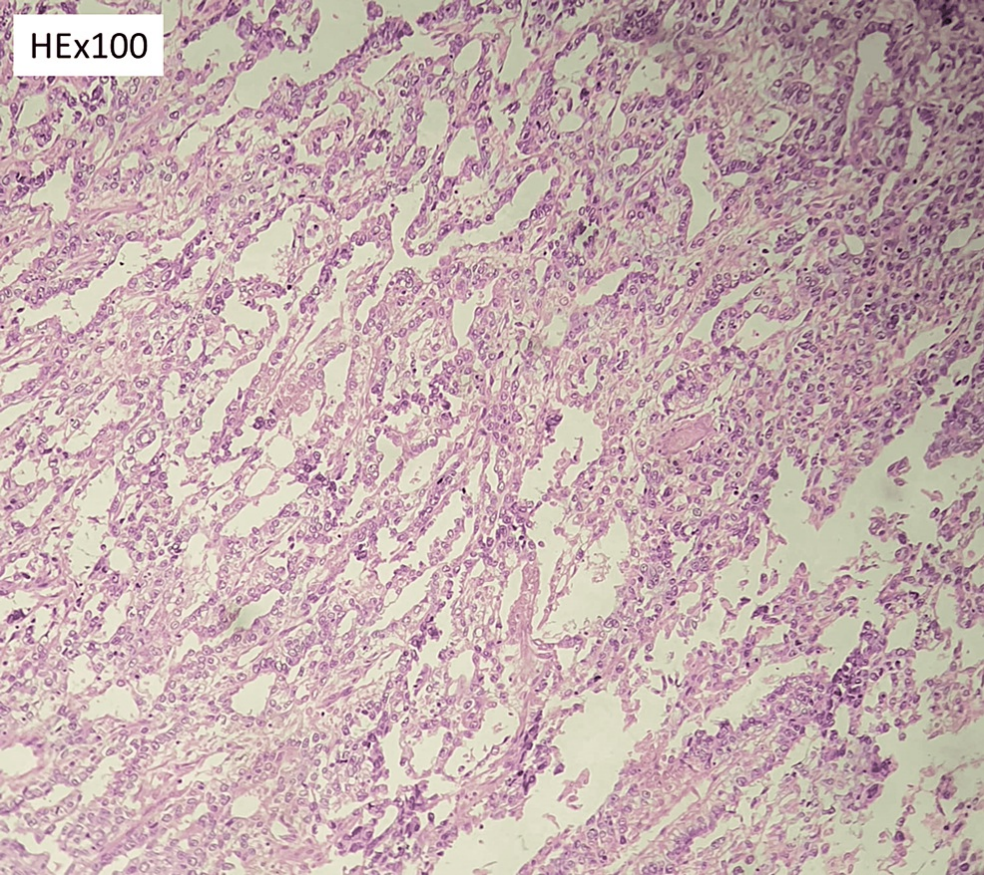
Microcystic pattern composed of anastomosing cords of flattened cells that form honeycomb like meshwork. Intraluminal spaces enclosing mucoid / basophilic material

**Figure 5:**
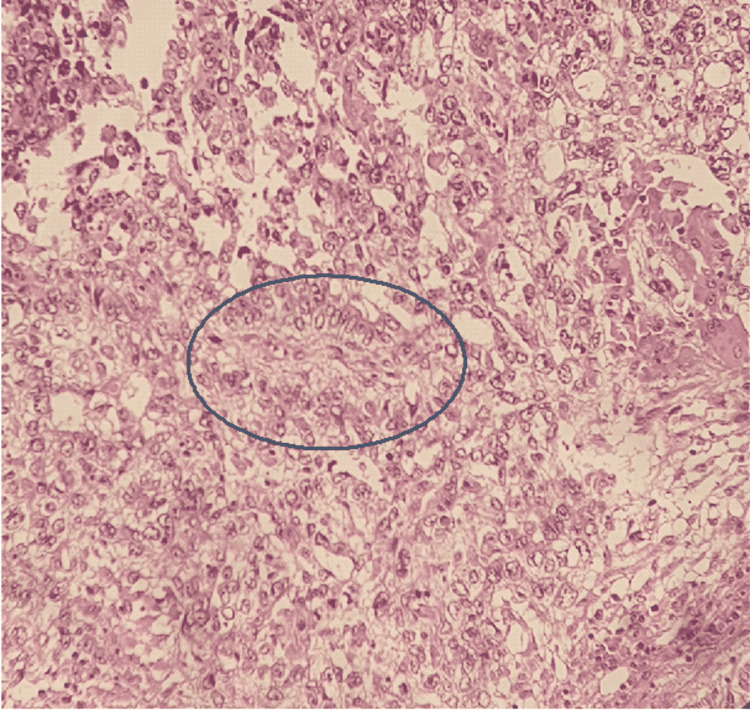
Tumor cells surrounding a central vessel (Schiller-Duval body). Hematoxylin & eosin staining.

**Figure 6:**
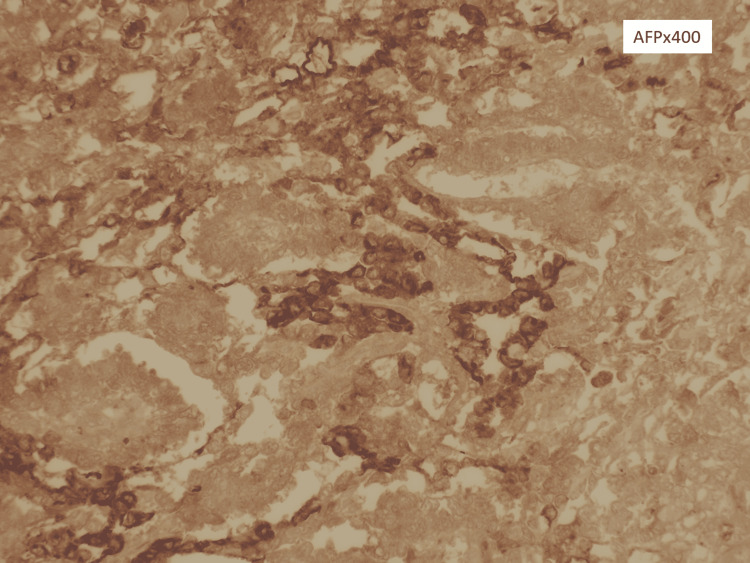
Strong and diffuse cytoplasmic expression of α-fetoprotein (AFP) from the neoplastic cells

**Figure 7:**
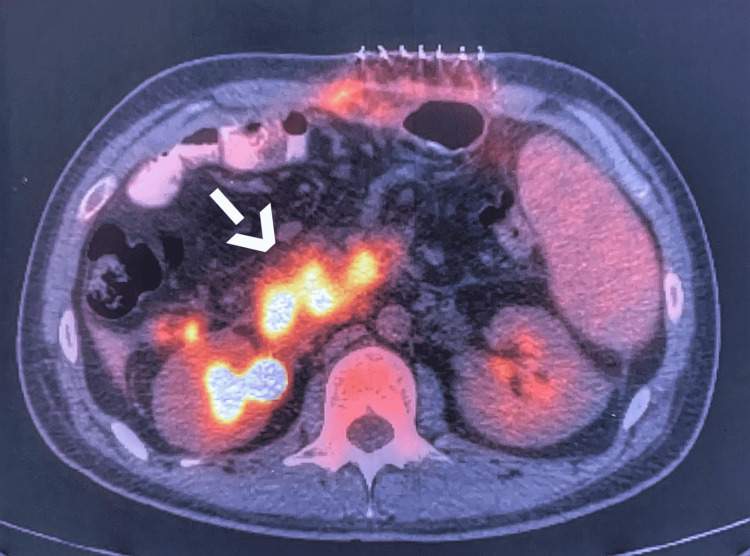
PET-CT image showing the pancreatic tumor (white arrow)

A 33-year-old male presented to our surgical department due to abdominal pain, vomiting, and weight loss. Blood tests showed obstructive jaundice (total bilirubin (TBIL): 17mg/dl, direct bilirubin (DBIL): 12mg/dl) and very high levels of α-fetoprotein (AFP>5845). Computed tomography revealed a 5cm mass of the head of the pancreas and on upper gastrointestinal endoscopy, an ulcerated lesion of the second part of the duodenum was discovered (Figure 1). Biopsies of this lesion indicated a pancreatic adenocarcinoma, infiltrating the duodenum near the Vater ampulla (Figures 2, 3). Since there was progressive biliary obstruction (TBIL: 30mg/dl), an exploratory laparotomy was decided and a standard pancreaticoduodenectomy (Whipple’s procedure) was performed. Histopathological examination of the specimen revealed cells with endodermal sinus pattern and characteristic Schiller-Duvall bodies, and cells with a reticular pattern (Figures 4, 5). Immunohistochemical staining of the cancer cells was positive for PLAP, AFP, CK8.18, and galectin-3 (Figure 6). Ki-67 was positive in 60-70% of the cells. As a result, the diagnosis of a yolk sac tumor was established. A preoperative scrotal ultrasound and a PET-CT scan had not revealed any primary lesion other than the pancreatic tumor (Figure 7). That being the case, the tumor was considered as a primary extragonadal (pancreatic) yolk sac tumor. Post-operative course was uneventful. The patient underwent eight cycles of adjuvant chemotherapy, based on cisplatin, and on follow-up there is no sign of recurrence, almost five years after the initial surgery.

## Discussion

Pancreatic GCTs, especially YSTs, are extremely rare. They often coexist with more common types of cancer, such as adenocarcinomas [6,7]. Genome analyses have shown that TP53 and KRAS are the most frequently mutated genes in the case of YSTs [7]. These tumors may reach a large size, with very few symptoms. A patient with extragonadal YST may present with abdominal pain or distention, back pain, anaemia, constitutional symptoms and even a palpable mass or vascular obstruction [3].

Because of their rarity, preoperative diagnosis of YSTs is difficult to determine. A significant rise in AFP levels in serum may be a clue for their diagnosis [4,6]. CT findings of YSTs often include large, smooth marginated, well enhancing, solid masses with a cystic, hemorrhagic or necrotic portion. However, due to their rarity there is no systematic study on YSTs’ CT features [5]. Endoscopic ultrasonography-guided fine needle aspiration could be an effective diagnostic modality for investigation of YSTs. Histopathology of the specimen provides definitive diagnosis. YSTs are identified by variable architectural patterns, including reticular, microcystic, papillary, and characteristic Schiller-Duvall bodies, which are composed of a monolayer of cubic or columnar neoplastic cells surrounding the capillaries, thin-walled blood sinus or small venus blood vessels [6]. Immunohistochemically, YSTs are characterized by a combination of low molecular weight cytokeratin (CK-8, CK-18), AE1/AE3, glypican 3, CD117, CD30, AFP, PLAP and OCT3/4. Sal-like protein 4 (SALL-4) may, also, be a sensitive diagnostic marker for YSTs [4].

Surgical excision with combined adjuvant chemotherapy is the treatment of choice. Chemotherapy has improved the survival of patients with YSTs, as these types of tumors are highly sensitive to cisplatinum-based protocols. However, many patients present with advanced local disease and distant metastasis. In this case, complete surgical excision is rarely feasible and the prognosis is poor [3,5].

## Conclusions

YST of the pancreas is a rare entity. High serum AFP levels could lead to an earlier diagnosis of an extragonadal YST. Physicians should be strongly aware of this type of tumor so that they may choose the optimal treatment when a YST is highly suspected.
